# Quantitative mining of wine aroma literature data for specification reuse

**DOI:** 10.1016/j.fochx.2026.104611

**Published:** 2026-10-15

**Authors:** Mei-Ling Yang, Hui Wang, Li-Xia Zhu

**Affiliations:** a College of Food Science and Engineering, Tarim University, Alar 843300, China; b Key Laboratory of Deep Processing of Southern Xinjiang Characteristic Agricultural Products, Tarim University, Alar 843300, China; c College of Food Science and Nutritional Engineering, China Agricultural University, Beijing 100083, China

**Keywords:** Wine aroma, Literature data mining, Machine learning, Varietal discrimination, Sensory validation

## Abstract

Wine aroma datasets are abundant yet hard to reuse due to dramatic cross-study differences in analytical platforms, quantification protocols and reporting formats. Quantitative volatile data published between 1998 and 2026 were curated and normalized for compound identities, concentrations, analytical metadata and odor thresholds. A total of 103 valid studies were screened from more than 5000 publications, and the dataset was constructed with records of 792 volatile compounds, 38 grape cultivars and samples from 22 countries. An initial panel of 31 exploratory candidate varietal markers was selected from the intersection of PCA, PLS-DA and random forest screening. A proof-of-concept evaluation used HS-SPME-GC–MS and sensory analysis of 24 commercial Cabernet Sauvignon and Marselan wines. The commercial-wine PLS-DA model yielded R^2^Y(cum) = 0.971 and Q^2^(cum) = 0.947, with an internal ROC-AUC of 0.840; no independent external test set was available. A framework for reusing literature-based wine aroma data was explored in this work. Incomplete metadata, inconsistent quantification approaches, missing-value handling and unbalanced varietal and geographical coverage remain limitations requiring independent validation under harmonized analytical conditions.

## Highlights

• A literature-derived database integrated volatile aroma profiles from 103 studies.
• Analytical provenance and missing-data patterns guided data harmonization.
• Multi-model chemometrics identified cultivar-associated volatile markers.
• Candidate markers were validated in Cabernet Sauvignon and Marselan wines.
• Cross-study heterogeneity remains a challenge for wine aroma data integration.

## 1 Introduction

Wine aroma represents a complex chemical and sensory property resulting from the combined effects of grape-derived precursors, microbial metabolism, maturation reactions, and interactions among volatile and non-volatile matrix components(Jiang et al., 2026; Li et al., 2025; Mango et al., 2018; Sam, Chen, Wang, Shi, & Tao, 2026). Over the past three decades, rapid advances in analytical technologies, including gas chromatography–mass spectrometry (GC–MS)(Sam et al., 2026), gas chromatography-olfactometry (GC-O)(Lyu, Tian, Ma, Xu, & Tang, 2025), comprehensive two-dimensional gas chromatography and high-resolution mass spectrometry(Koljani, Gomes, & pánik, 2023), have enabled the identification and characterization of thousands of volatile compounds in wines. These studies have greatly expanded our understanding of wine aroma formation and provided essential scientific support for wine quality evaluation, authenticity assessment, and industrial development(Styger, Prior, & Bauer, 2011a; Welke, Hernandes, Nicolli, & Aith.Barbará, J., Biasoto, A. C. T., & Zini, C. A., 2021). Recent studies have integrated advanced chromatographic platforms with chemometric and machine-learning approaches to achieve high-resolution aroma profiling, varietal authentication, and sensory prediction(Athanasiadis & Ioannides, 2021). For example, GC × GC coupled with high-resolution mass spectrometry combined with machine-learning algorithms such as random forest and support vector machines has successfully identified geographical and varietal discrimination markers, and demonstrated promising performance in predicting sensory attributes from volatile profiles(Salopek et al., 2026). Bibliometric analysis provides an effective approach for systematically evaluating research development by revealing publication trends, geographical distribution, and knowledge evolution(Du, Ye, Hao, Tan, & Ye, 2024). When combined with chemometric analysis and machine-learning algorithms, literature-derived datasets may provide new opportunities for identifying stable aroma patterns and developing transferable molecular markers(Cosme, Vilela, Oliveira, Aires, & Pinto, 2025). These wine authentication and chemometric studies are generally based on datasets generated under controlled experimental designs, where samples are analyzed using identical protocols(Koljani, Gomes, & pánik, I., 2023). However, these advances also highlight the increasing potential of data-driven strategies for objective evaluation and intelligent management of global food systems. (Kirtil, 2025).

However, despite the rapid accumulation of wine aroma research, quantitative aroma data remain highly fragmented and difficult to integrate across independent studies. They cannot address whether measurements generated by different laboratories, analytical platforms, and experimental conditions can be reliably integrated. Published datasets differ substantially in extraction procedures, analytical platforms, identification criteria, quantification strategies, and reporting formats(Pati et al., 2021). Compound concentrations are often reported as absolute concentrations, relative abundances, or surrogate-based estimates, while non-detected, below-quantification, unmeasured, and unreported compounds are often inconsistently defined. Furthermore, odor thresholds vary depending on matrix composition and sensory methodology, and complex interactions among aroma compounds may result in synergistic, masking, or inhibitory effects(Hu et al., 2026; Song et al., 2026). Therefore, direct comparison and secondary utilization of literature-derived aroma data remain challenging, and statistical normalization alone cannot fully eliminate study and platform-specific heterogeneity(Ziegler et al., 2019), while comprehensive frameworks integrating literature mining, database construction, statistical modelling, and experimental validation remain limited. Consequently, the establishment of a standardized, expandable, and quality-controlled wine aroma database is essential for enabling robust cross-study analysis and supporting future applications of artificial intelligence and machine learning in wine research(Gomis-Bellmunt, Claret, Guerrero, & Pérez-Elortondo, 2024).

Therefore, this study aimed to construct and critically evaluate a literature-derived wine aroma database by integrating global quantitative aroma datasets. Specifically, the objectives were to: (i) systematically collect and harmonize published quantitative volatile compound data with detailed analytical provenance; (ii) characterize global trends and factors influencing wine aroma variation, including grape cultivar, geographical origin, wine type, and analytical methodology; (iii) identify cultivar-associated aroma markers using integrated chemometric approaches including PCA, PLS-DA, and RF models; and (iv) explore the potential applicability of the identified markers using commercial Cabernet Sauvignon and Marselan wines through a proof-of-concept HS-SPME-GC–MS analysis and sensory evaluation. This study provides a framework for understanding global wine aroma diversity, improving compound discrimination, and facilitating future intelligent applications in wine quality assessment.

## 2 Materials and methods

### 2.1 Literature search and wine aroma database construction

A systematic literature search was conducted to establish a wine aroma compound database, following the Preferred Reporting Items for Systematic Reviews and Meta-Analyses (PRISMA) guidelines for systematic search and reporting(Gallego-Valero, Moral-Pajares, & García-Moral, 2026). Web of Science Core Collection, Google Scholar, Scopus, PubMed, and ScienceDirect were searched for peer-reviewed studies on wine volatile aroma compounds published from 1 January 1998 to 30 January 2026; the final search was performed on 30 January 2026. The search strategy combined keywords using Boolean operators: within each concept group, synonyms were connected with “OR”, and cross-group terms were connected with “AND”. The search was restricted to titles, abstracts, and keywords where supported, and limited to peer-reviewed original articles written in English. The detailed screening process and the number of records at each stage are reported in the Results section (Section 3.1).

The database-specific search strings were as follows. Web of Science: TS = ((“wine aroma” OR “wine aromas” OR “wine volatile compound” OR “wine volatile profile” OR “wine flavor compound” OR “wine flavour compound”) AND (quantitative OR concentration OR content OR profiling) AND (“gas chromatography” OR GC–MS OR GC-O OR HS-SPME OR SPME OR “solid phase extraction” OR “liquid-liquid extraction”)). Scopus: TITLE-ABS-KEY((“wine aroma” OR “wine aromas” OR “wine volatile compound” OR “wine volatile profile” OR “wine flavor compound” OR “wine flavor compound”) AND (quantitative OR concentration OR content OR profiling) AND (“gas chromatography” OR GC–MS OR GC-O OR HS-SPME OR SPME OR “solid phase extraction” OR “liquid-liquid extraction”)). PubMed: ((“wine aroma”[Title/Abstract] OR “wine aromas”[Title/Abstract] OR “wine volatile compound”[Title/Abstract] OR “wine volatile compounds”[Title/Abstract] OR “wine volatile profile”[Title/Abstract]) AND (quantitative[Title/Abstract] OR concentration[Title/Abstract] OR profiling[Title/Abstract]) AND (“gas chromatography”[Title/Abstract] OR GC–MS[Title/Abstract] OR GC-O[Title/Abstract] OR HS-SPME[Title/Abstract] OR SPME[Title/Abstract])). ScienceDirect: ((“wine aroma” OR “wine volatile compounds” OR “wine volatile profile”) AND (quantitative OR concentration OR profiling) AND (“gas chromatography” OR GC–MS OR GC-O OR HS-SPME OR SPME)). Google Scholar was searched using the combinations “wine aroma” volatile compounds quantitative GC–MS; “wine volatile profile” concentration HS-SPME; and “wine aroma compounds” quantitative GC-O.

Studies were included when they (i) investigated grape wines, (ii) reported identified volatile aroma compounds with quantitative or semi-quantitative data, and (iii) provided sufficient information on grape cultivar, wine characteristics, and analytical methods. Studies involving only grape berries, non-wine beverages, qualitative compound lists, or duplicated datasets were excluded. Records were imported into EndNote for deduplication before screening. Review articles were used only for citation tracking, not quantitative data extraction. Full-text exclusions also included insufficient cultivar, sample or analytical information and inaccessible full text. Of 300 articles assessed in full, 197 were excluded and 103 were included.

After title/abstract screening and full-text evaluation, eligible studies were selected for database construction. Information including grape cultivar, geographical origin, wine type, analytical platform, extraction method, compound identity, concentration, and odor threshold was extracted. Compound names were standardized using chemical identifiers, such as CAS numbers and PubChem information when available.

Odor-threshold values were extracted with their units, source references and available matrix descriptions. A uniform model-wine-only restriction was not imposed, and thresholds from different sources were not averaged. Where multiple values were available, a value for wine or the closest available wine matrix was selected. Thresholds were converted to μg/L only when the conversion basis was explicit; compounds without a traceable numerical threshold were recorded as NR. Compound-specific source records are identified in Supplementary Table S1 and Supplementary Tables S2 and S4.

Concentrations reported in different units were converted to μg/L when the original basis was clearly defined. Records with ambiguous units or insufficient quantitative information were excluded from quantitative analyses. Accepted absolute units were ng/L, μg/L and mg/L; values in ng/L were divided by 1000 and values in mg/L were multiplied by 1000. Relative peak areas, percentages and surrogate-standard estimates were retained with their original quantification basis and were not considered equivalent to absolute concentrations. Normalization does not establish equivalence among these measurement types. No included article was excluded solely because it used a different concentration unit, but a separate record-level log of values excluded for ambiguous units was not retained; the exact number cannot be reconstructed without re-extraction. The extracted data were organized into a structured database, with each record representing the volatile aroma profile of an individual wine sample or sample group.

### 2.2 Data harmonization and missing value handling

Due to differences in analytical platforms, extraction procedures, detection capabilities, quantification strategies, and reporting practices across independent studies, heterogeneous data structures were frequently encountered during database construction. To improve cross-study comparability, the extracted data were harmonized with respect to compound identity, chemical classification, concentration units, and analytical metadata. Whenever sufficient information was available, compound names were manually curated and standardized using CAS numbers, PubChem records, and published chemical information. Concentration values were converted to μg/L only when the original units and the corresponding conversion basis were clearly defined. Information on extraction methods, instrumental platforms, compound identification approaches, and quantification strategies was retained and recorded as analytical metadata.

In the revised datasets, entries without available quantitative values were denoted as “NR” with unavailable values potentially representing compounds that were not analyzed, not detected, below the limit of quantification (LOQ), not reported, or reported without reliable quantitative information. These annotations reflect the original data availability and should not be interpreted as evidence of compound absence. Available source reporting information was retained where possible, because compounds that were not reported or quantitatively analyzed could not be reliably interpreted as absent or assigned a concentration of zero in wine samples. However, for multivariate statistical analyses (e.g., PCA and PLS-DA), which require a complete data matrix without missing values, entries lacking reported quantitative concentrations were numerically coded as zero solely for computational purposes. This coding strategy was applied only to facilitate dimensionality reduction and pattern recognition and should not be interpreted as evidence of experimental absence or zero concentration of the corresponding compounds. Similar considerations regarding the interpretation of non-detected or missing analytical signals have been discussed previously(Davis, Firzli, Keppler, Richardson, & Bean, 2025). This approach was selected to avoid introducing artificially estimated concentrations into the large-scale heterogeneous literature dataset. Nevertheless, because zero encoding may partly reflect differences in analytical coverage among studies, analytical metadata were retained and considered when interpreting cross-study patterns.

For a missing-data sensitivity analysis, entries coded as zero solely because they were unavailable or unreported were recoded as missing and imputed in R using k-nearest neighbours (k = 5). PCA and PLS-DA were rerun using the same downstream modelling framework. This sensitivity analysis does not recover the true unobserved concentrations, because source studies did not consistently distinguish analytical non-coverage, non-detection and below-quantification observations.

To reduce the influence of highly sparse variables, compounds with insufficient reporting frequency or inadequate quantitative support were excluded or interpreted cautiously during downstream analyses. Marker identification was therefore focused on compounds supported by available quantitative observations and showing consistent contributions across the integrated multivariate models.

Sensory descriptors were harmonized using a controlled vocabulary adapted from the Wine Aroma Wheel. Original terms were retained, while spelling and lexical variants were standardized and specific descriptors were mapped to broader categories: blackcurrant, berry, red fruit and cherry to fruity; flower, dried flower and violet to floral; and green pepper and herbaceous to vegetal/green. Thus, berry-like and strawberry could share a higher-level category without being treated as exact synonyms. Ambiguous terms were retained without forced assignment, and taste or mouthfeel terms were excluded from aroma-category mapping. This was a rule-based harmonization scheme, not a formal computational ontology.

### 2.3 Database characterization and aroma compound profiling

The constructed database was first characterized to evaluate the temporal, geographical, and chemical distribution of wine aroma research. The publication trends, geographical coverage of grape cultivars, and distribution of major aroma compound classes were visualized using R software.

The geographical distribution of reported grape cultivars was illustrated using global and regional maps. The chemical composition characteristics of the database were summarized according to major volatile compound categories, including esters, higher alcohols, terpenes, volatile acids, aldehydes, ketones, and other aroma-related compounds.

To investigate the contribution of different factors to wine aroma variation, aroma profiles were compared according to grape variety, geographical origin, wine type, and analytical methodology. These comparisons described factor-associated patterns without joint adjustment for geography, wine type, analytical method or study identity; subsequent chemometric analyses focused primarily on varietal discrimination and marker compound identification.

### 2.4 Chemometric analysis and marker compound identification

To identify key differential compounds, aroma-related chemical information was first extracted from the literature across four dimensions, including geographical origin, wine type, grape variety, and extraction method. All datasets were normalized using the Min-Max method in Origin 2024(OriginLab, Northampton, MA, USA), scaling each variable to a range of 0–1. For a nonconstant compound variable, Min–Max normalization uses x_MM = (x − x_min)/(x_max − x_min), whereas *Z*-score standardization uses x_Z = (x − μ)/s, where μ and s denote its mean and standard deviation. Both are affine transformations that shift and rescale each variable, producing dimensionless values and preserving the ordering of observations within that variable. They therefore serve a similar purpose in reducing numerical scale differences. However, Min–Max uses the observed range, whereas *Z*-score uses the standard deviation; they do not necessarily yield identical variable weights or multivariate results. Min–Max normalization was the scaling procedure used here; neither a separate Z-score step nor ComBat batch correction was applied.(Awale, Liu, & Kwasniewski, 2021) Subsequent analyses focused on varietal discrimination and candidate-marker identification; this focus does not establish grape variety as independently dominant over the other factors.

Three statistical models were subsequently established in sequence based on the clean datasets. PCA was first performed using compounds with OAV > 1 (concentration of a compound divided by its corresponding odor threshold value) across all grape varieties to evaluate the natural clustering and separation trends among different samples through score plots. To further conduct supervised classification and variable selection, PLS-DA was employed, with grape variety set as the Y variable and aroma compound concentrations used as the X variables. Model performance was assessed using R^2^X, R^2^Y and Q^2^, with permutation tests (*n* = 200) used to assess the risk of overfitting. Compounds with variable importance in projection (VIP) values >1 were subsequently selected as key differential compounds in the preliminary screening stage.

In addition, an RF classification model was established(Ranaweera, Capone, Bastian, Cozzolino, & Jeffery, 2021; Yavas, Kim, Chen, Kadlec, & Ji, 2025) and the Increase in Mean Squared Error (%IncMSE, permutation importance) was employed to evaluate variable importance. Compounds ranked within the top 50% in importance were selected as the key variables identified by the RF model. By integrating the results obtained from the three statistical models, only compounds simultaneously meeting the PCA criterion (absolute loading >0.5), the PLS-DA criterion (VIP > 1) and the RF criterion (top-50% importance ranking) were retained. The final panel was the intersection of these three sets. A total of 31 compounds met all three criteria and were retained for exploratory evaluation. The upper-50% RF cutoff was a rank-based screening rule and was not optimized against classification accuracy.

The RF analysis used the randomForest package in R with 63 samples, six classes and 80 compound variables. A stratified 49/14 partition was defined, and tuning used five-fold stratified cross-validation within the training data. The selected settings were 100 trees, mtry = 9 and nodesize = 1. Balanced bootstrap samples were drawn using the smallest class size. After tuning, the model was refitted to all 63 samples for out-of-bag (OOB) evaluation and variable ranking. These internal estimates do not constitute independent external validation.

### 2.5 Proof-of-concept experimental evaluation

To explore the potential applicability of the identified varietal markers, a proof-of-concept experimental evaluation was conducted using commercial wine samples.

#### 2.5.1 Wine samples

Two representative red wine grape cultivars, Cabernet Sauvignon and Marselan, were selected for the proof-of-concept evaluation because they were among the most frequently investigated varieties in the literature-derived database and showed distinct aroma characteristics (Ángela, Emilia, & Sandra, 2022).

The collection of 24 commercial dry red wines, including 12 Cabernet Sauvignon and 12 Marselan samples from vintages 2020–2022, represented major producing regions across China. All samples were stored under controlled conditions before analysis.

#### 2.5.2 HS-SPME-GC–MS analysis

The volatile compounds of all wine samples were analyzed by HS-SPME-GC–MS following a previously reported method(Yang et al., 2026). For sample preparation and HS-SPME extraction, 8 mL of wine sample was transferred into a 20 mL headspace vial, and 1.5 g of NaCl was added. Before sealing, 10 μL of 4-methyl-2-pentanol internal-standard solution was added. The vial was sealed and equilibrated in a water bath at 50 °C for 10 min. A 50/30 μm DVB/CAR/PDMS fiber was then exposed to the headspace at 50 °C for 40 min for volatile extraction, followed by thermal desorption in the injector at 250 °C for 5 min. Gas chromatographic separation was performed on an Agilent Technologies HP-INNOWax capillary column (60 m × 0.25 mm × 0.25 μm). The injector temperature was set at 250 °C and operated in split mode with a split ratio of 5:1. Helium (He) was used as the carrier gas at a flow rate of 1.0 mL/min. The oven temperature program was as follows: initial temperature of 50 °C for 1 min, increased to 220 °C at 3 °C/min and held for 3 min, and then increased to 240 °C at 10 °C/min. Mass spectrometric analysis was carried out using electron ionization (EI) at 70 eV. The ion source, quadrupole, and transfer line temperatures were set at 230, 150, and 250 °C, respectively. Data were acquired in full-scan mode over a mass range of *m*/*z* 29–300, with a solvent delay of 3.0 min. Compound identification was achieved by comparison with mass spectral libraries, retention indices, and authentic standards, while compounds with authentic standards were quantified using compound-specific calibration curves and other compounds were estimated semi-quantitatively relative to 4-methyl-2-pentanol(Ortega, López, Cacho, & Ferreira, 2001). Retention indices, standards, calibration equations, ranges and quantification categories are reported in Supplementary Table S6. Calibrated concentrations fell within the respective working ranges. Compound-specific LOD/LOQ and spike recovery were not determined, so the procedure is not a fully validated quantitative method.

### 2.6 Sensory evaluation

A panel of 10 trained assessors (5 males and 5 females, aged 24–38 years) with prior experience in wine sensory evaluation was recruited for the sensory analysis. Before formal evaluation, all assessors participated in three training sessions focused on aroma descriptor recognition, intensity scaling, and evaluation standardization(Stefanowicz, 2013). Representative reference standards were used to familiarize assessors with the six target sensory attributes, including fruity, floral, caramel, spicy, green, and animal-like notes(Caporaso, Whitworth, Cui, & Fisk, 2018). During training, consensus discussions were conducted to improve descriptor consistency and scoring reproducibility among panel members.

Reference standards were ethyl laurate (5 mg/L) and isobutyl acetate (2 mg/L) for fruity notes; geraniol (0.5 mg/L) and linalool (0.1 mg/L) for floral notes; 2,3-butanediol (50 mg/L) and ethyl caprate (5 mg/L) for caramel; decanoic acid (5 mg/L) and isovaleric acid (1 mg/L) for animal; 1-hexanol (5 mg/L) for green/herbaceous; and isoamyl alcohol (50 mg/L) as the operational spicy/pungent reference. Each was prepared separately in 12% (*v*/v) ethanol with 4–5 g/L tartaric acid, adjusted to pH 3.3–3.5, and equilibrated for 24 h

Sensory evaluation was performed in individual sensory booths under controlled environmental conditions. Wine samples were presented in randomized order and evaluated using a structured 0–5 intensity scale. Each sample was independently evaluated in duplicate to improve reproducibility. The 24 wines were assessed over three sessions separated by at least 24 h, with eight wines and 16 presentations per assessor per session. Duplicate presentations were independently randomized, balanced and non-consecutive. Sessions lasted approximately 45–60 min, including a 10–15 min rest between blocks. Each presentation comprised 30 mL at 20 °C in a covered ISO glass with a randomized three-digit code; samples were separated by 2–3 min.

To assess panel reliability and repeatability, analysis of variance (ANOVA) was conducted to evaluate assessor consistency and sample discrimination ability. In addition, panel performance and repeatability were examined using F-values and PanelCheck-based consistency assessment, following previously reported approaches (Cosme et al., 2025; Piombino et al., 2026). Attribute-specific ANOVA, discrimination, MSE and RV results are reported in Supplementary Tables S5A–S5C.

### 2.7 Statistical analysis

Data organization and preliminary processing were performed using Microsoft Excel. Statistical analyses were conducted using IBM SPSS Statistics and Origin 2024. Advanced visualization, including global distribution maps and circular stacked bar plots, was performed using R software (version 4.3.3) with the packages tidyverse, ggplot2, viridis, maps, scatterpie, rnaturalearth, and rnaturalearthdata.

For the grape-variety comparison, total volatile concentration was the sum of reported compound concentrations per record. One nonnumeric entry was excluded from 461 records, leaving 460 records across 38 groups (*n* = 2–77; median = 5). Levene and Brown–Forsythe tests assessed variance homogeneity. Welch's one-way ANOVA was the primary test; conventional ANOVA and η^2^ and ω^2^ effect sizes were also reported. Pairwise Welch tests used Holm adjustment. Geography, wine type, analytical method and study identity were not jointly controlled. Multivariate analyses including PCA, PLS-DA, and RF were performed for sample classification and marker compound identification.

PLS-DA model performance was evaluated using R^2^X, R^2^Y, Q^2^, and permutation testing. Statistical significance was defined as *p* < 0.05.

## 3 Results and analysis

### 3.1 Aroma database construction

A total of 5763 English-language publications related to wine aroma were initially identified through searches of Web of Science, Google Scholar, Scopus, PubMed, and ScienceDirect, as shown in Fig. 1. Following duplicate removal (3700 records remaining), title and abstract screening (1300 records remaining), and full-text assessment (300 articles remaining), 103 eligible studies were included in the final dataset. These studies encompassed quantitative data for 792 volatile aroma compounds across 38 grape cultivars from 22 countries (Supplementary Table S1). The 792 volatile aroma compounds comprised 200 alcohols, 68 aldehydes, 66 acids, 267 esters, 80 ketones, 19 terpenes, and 92 other compounds. In terms of country frequency, China appeared 35 times, Spain 19, Australia 9, Brazil 5, Turkey 4, Southern Africa 3, Croatia 3, Italy 3, Uruguay 3, USA 3, France 2, Romania 2, Serbia 2, North Macedonia 2, and the remaining 8 countries collectively 8 times. Regarding grape variety frequency, the most frequently studied varieties were Cabernet Sauvignon (31 times), Merlot (22), Chardonnay (14), Muscat (10), Marselan (10), and Shiraz (8), showing substantial concentration of the available literature in these six cultivars. Overall, this comprehensive dataset provides a robust foundation for subsequent database construction and comparative analysis of wine aroma profiles. Furthermore, the database contains comprehensive information for each compound, including odor threshold values, molecular formulas, and molecular weights. Among the 792 aroma compounds identified from wine-related studies, odor threshold values were available for 191 compounds, whereas thresholds for the remaining 601 compounds were not reported in the reviewed literature. Therefore, these compounds were recorded as NR (Not Reported) in Supplementary Table S2. Importantly, NR indicates that no odor threshold value was identified within the collected literature sources and does not imply the absence of aroma activity or the inability of the compound to contribute to wine aroma. Threshold-missing compounds were excluded from OAV-based screening because a usable OAV could not be calculated.

**Fig. 1:**
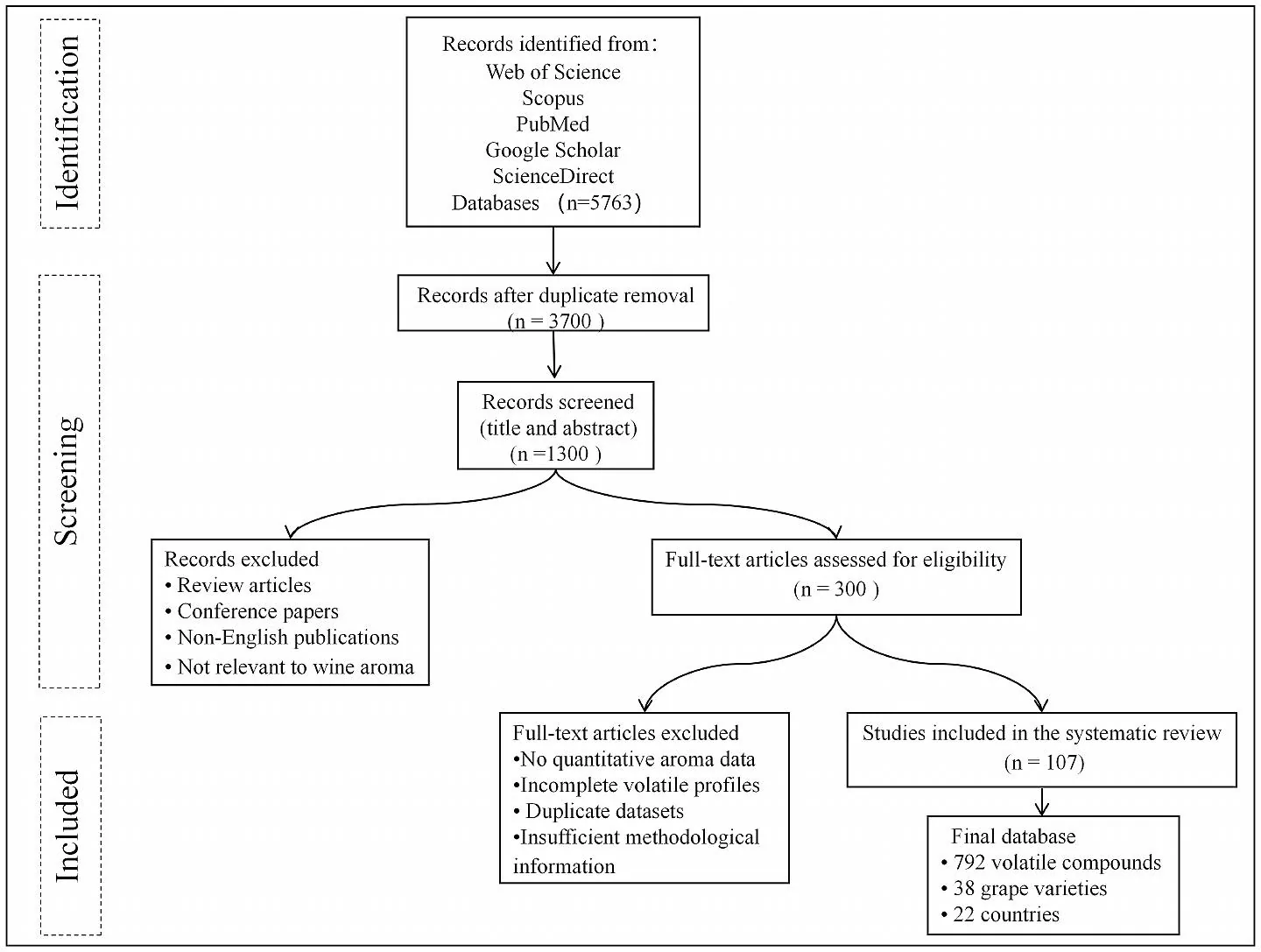
PRISMA flow diagram illustrating the literature search and study selection process.

Using the reported country counts and 103-study denominator, China accounted for 34.0%, Spain for 18.4% and Australia for 8.7% of included studies, together representing 61.2%. The cultivar frequencies in Fig. 2, Fig. 4 likewise indicate unequal research coverage. These distributions may reflect publication and analytical attention as well as biological differences.

**Fig. 2:**
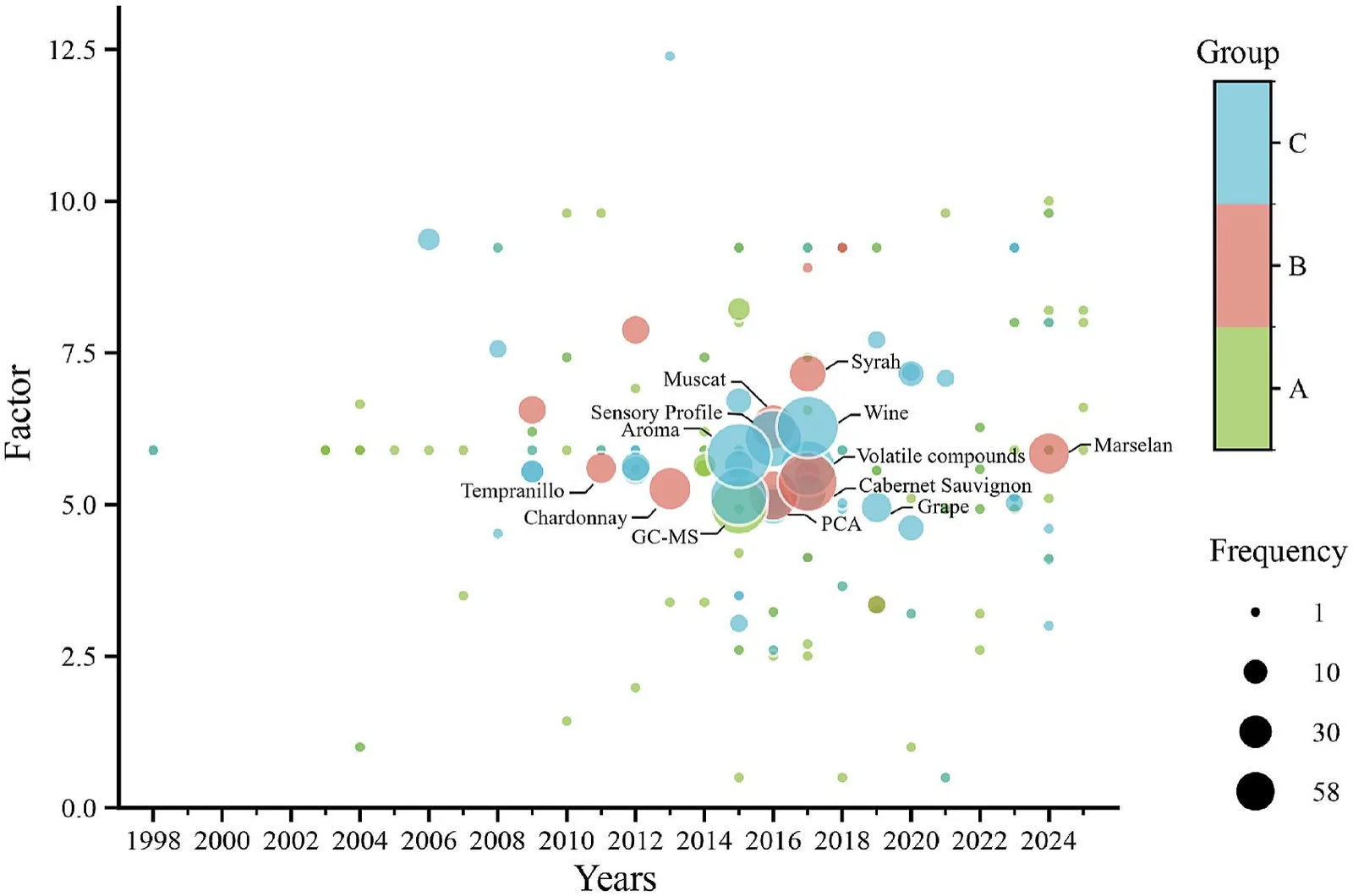
Bubble plot illustrating the results of bibliometric analysis. *Note:* Bubble colours represent three key dimensions: (A) first author; (B) grape varieties investigated; and (C) keywords reported in the literature.

### 3.2 Bibliometric characteristics and research coverage of wine aroma studies

#### 3.2.1 Temporal and keyword trends

The temporal distribution and occurrence frequency of first authors, grape cultivars, and research keywords across the 103 included studies are shown in Fig. 2. Green bubbles represent first authors and are scattered across different publication years and journal impact factor ranges. Most are small or medium in size, with no single author or research group showing a dominant contribution, indicating that the included studies were conducted by a wide range of independent research teams rather than being led by a few major contributors.

Red bubbles in Fig. 2 indicate different grape cultivars and their occurrence frequency increased markedly after 2010, with the highest concentration observed between 2014 and 2024. Among them, Cabernet Sauvignon, Chardonnay, Tempranillo, Merlot, Marselan, Syrah, and Muscat are represented by substantially larger bubbles than other cultivars, indicating that these cultivars were investigated more frequently. Moreover, the larger bubbles are predominantly distributed in recent years, suggesting a marked increase in cultivar-related studies after 2014.

Research keywords indicated by Blue bubbles were also mainly distributed after 2010. The keyword *wine* showed the highest occurrence frequency, whereas aroma, volatile compounds, GC–MS, PCA, and sensory profile appeared repeatedly in recent years, reflecting the most frequently investigated topics in this field. Overall, studies published after 2010, particularly those from 2014 to 2024, showed a substantial increase in both cultivar-related research and the application of analytical approaches, highlighting the growing emphasis on varietal aroma characteristics and advanced analytical techniques.

#### 3.2.2 Literature occurrence frequency of aroma compounds

To evaluate the research coverage and reporting characteristics of individual aroma compounds within the established database, the literature occurrence frequency of each volatile compound was calculated based on the number of independent records in which the compound was reported. Among the 792 identified aroma compounds, occurrence frequencies showed substantial variation, indicating a highly uneven distribution of research attention and reporting coverage among individual compounds. The mean occurrence frequency was 20.89, whereas the median value was only 5, with the most frequently reported compound appearing in 377 independent records.

The large difference between the mean and median values, together with the positive skewness value (4.47), indicated a strongly right-skewed distribution. A limited number of well-characterized aroma compounds accounted for a considerable proportion of literature records, whereas most compounds were reported only occasionally.

To visualize this highly skewed distribution, occurrence frequencies were transformed using log10(Frequency + 1). As shown in Fig. 3, most compounds were concentrated within the lower-frequency range, whereas only a small subset showed extensive literature coverage. These highly reported compounds likely represent classical aroma markers that have received long-term attention due to their sensory importance, analytical accessibility, or established association with wine quality. Conversely, compounds with low occurrence frequencies may represent emerging aroma candidates, compounds with limited analytical detection, or insufficiently investigated chemical components.

**Fig. 3:**
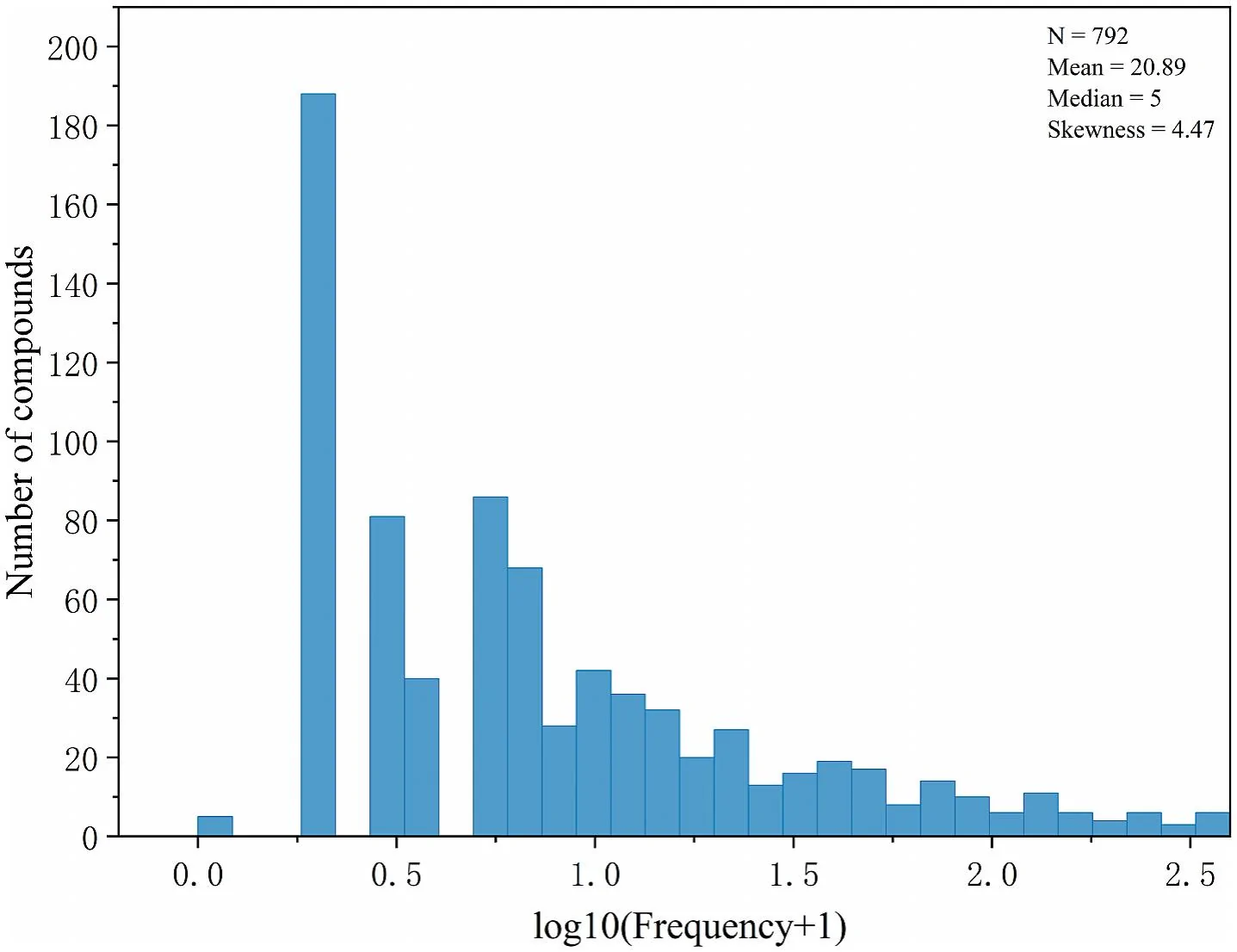
Research coverage distribution of wine aroma compounds based on literature occurrence frequency.

This uneven research distribution highlights an important challenge for literature-derived aroma databases: current knowledge is strongly influenced by historically studied compounds, whereas the potential contribution of less frequently reported compounds remains insufficiently explored.

### 3.3 Geographical distribution

The 103 selected studies on aroma compounds involved 22 countries and 38 grape cultivars (Fig. 4A). The geographical distribution of research was highly uneven, with most studies originating from China, European countries, Australia, and the United States, whereas relatively few were conducted in South America and Africa. In Europe, Spain exhibited the greatest cultivar diversity, covering more than 17 grape cultivars, including Verdejo, Tempranillo, Chardonnay, and Cabernet Sauvignon. Among these, Tempranillo (18%), Chardonnay (9%), and Cabernet Sauvignon (9%) were the most frequently investigated. Studies from Southeastern Europe (Serbia, Croatia, Montenegro, and North Macedonia) primarily focused on Muscat cultivars. In Asia, China accounted for the largest number of studies, with Cabernet Sauvignon accounting for 33% of all domestic investigations. In South America, research was concentrated in Chile, Brazil, and Uruguay, with Brazil being the most active contributor. The cultivars investigated in Brazil included Syrah, Merlot, Chardonnay, BRS Violeta, and BRS Carmem. In North America, studies from the United States focused mainly on Muscat and Merlot, whereas only one cultivar was reported in Canada. Studies from Australia investigated six cultivars, including Syrah, Merlot, Grenache, Chardonnay, and Cabernet Sauvignon.

**Fig. 4:**
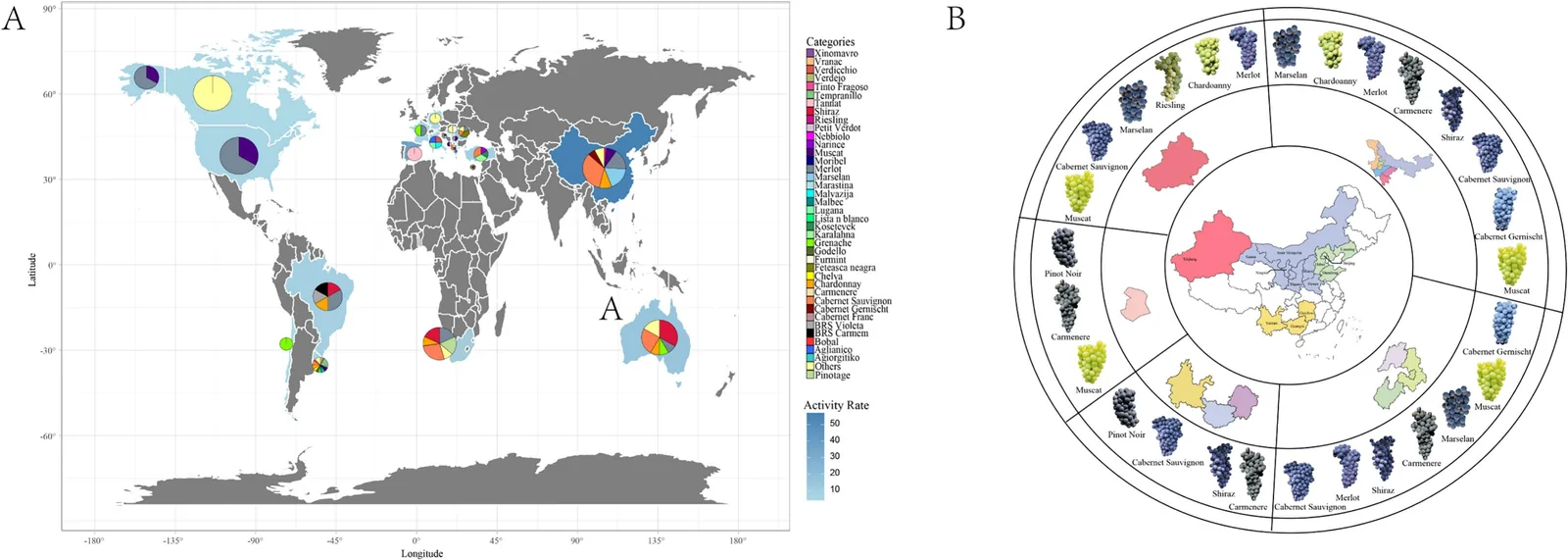
Geographical distribution of grape varieties in wine aroma studies. (A) Global distribution of grape varieties; (B) distribution of grape varieties in China. *Note:* Pie charts indicate the proportion of grape varieties investigated in each country, while the colour intensity of the background reflects the research frequency.

Within China, Fig. 4B shows that research was primarily concentrated in Ningxia (31%), Hebei (21%), Xinjiang (14%), and Shanxi (9%), while Yunnan (2%), Guangxi (2%), Liaoning (2%), and other regions contributed comparatively fewer studies. Research in Ningxia and Xinjiang mainly focused on international cultivars, particularly Cabernet Sauvignon, Chardonnay, and Marselan. In contrast, studies from Hebei and Shanxi covered a broader range of cultivars, including Cabernet Sauvignon, Marselan, and Merlot. Only Cabernet Sauvignon was investigated in studies from Guangxi and Yunnan.

### 3.4 Qualitative and quantitative visualization analysis of aroma compounds

To further systematically investigate the key factors influencing wine aroma compounds, quantitative and qualitative visualization analyses were performed based on the dataset of 103 studies. Differential analyses were conducted from four dimensions, including grape variety, wine type, production region, and analytical method (Fig. 5A). For grape variety, the Brown–Forsythe test indicated unequal variances (F(37, 422) = 2.429, *p* < 0.001). Welch's ANOVA showed an association with total volatile concentration (F(37, 36.721) = 328.900, *p* < 0.001). Conventional ANOVA yielded F(37, 422) = 2.463, *p* < 0.001, with η^2^ = 0.178 and ω^2^ = 0.105. These unadjusted comparisons do not establish grape variety as independently dominant over geography, wine type or analytical method.

**Fig. 5:**
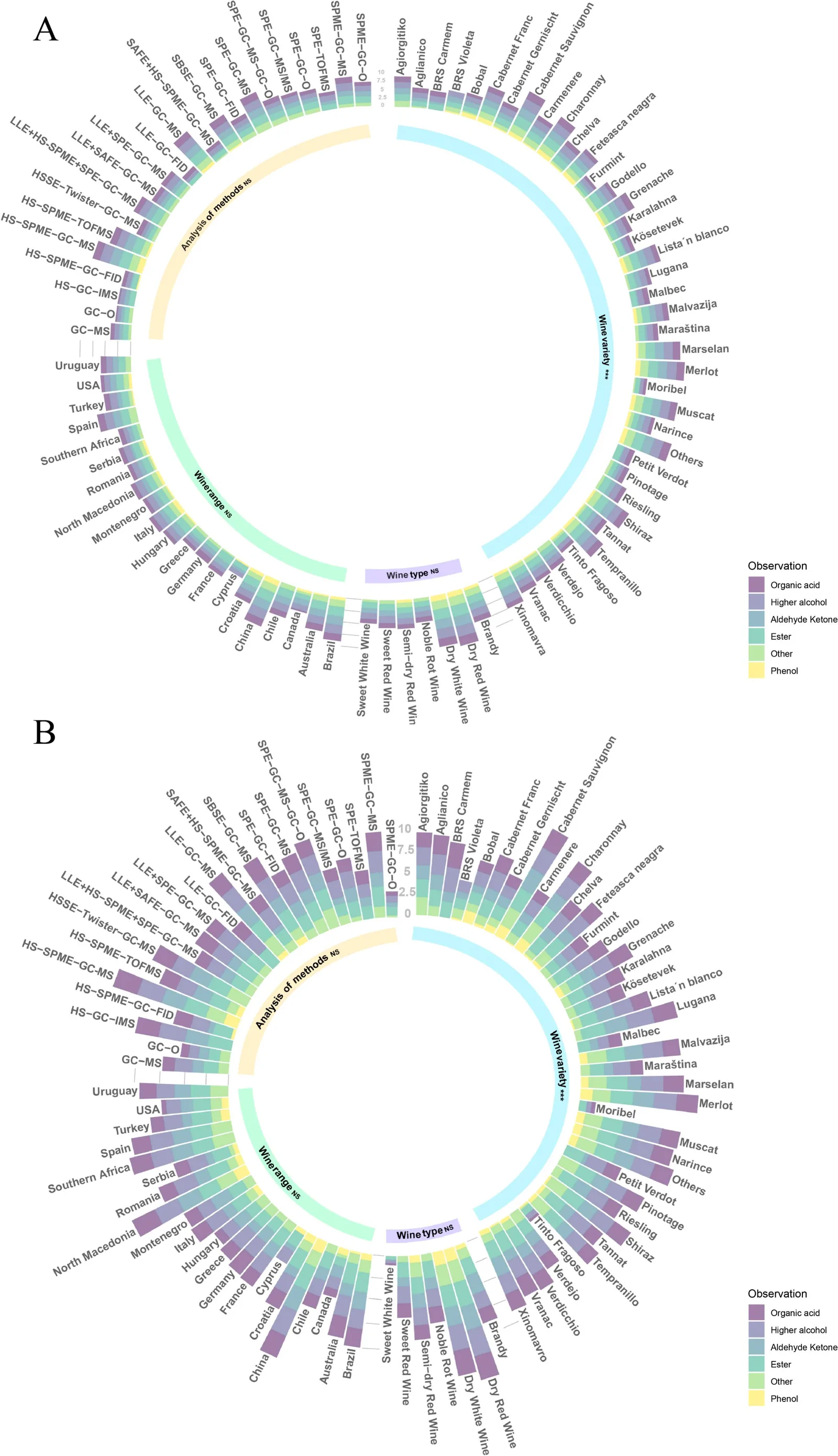
Qualitative and quantitative visualization of wine aroma compounds. (A) Qualitative visualization of aroma compounds; (B) quantitative visualization of aroma compounds.

Distinct differences in aroma composition and compound concentration were observed among grape varieties. Among the 38 grape varieties analyzed, Merlot and Cabernet Sauvignon exhibited the highest total concentrations of volatile compounds. In particular, 22 varieties were clearly identified as having different observed compositional profiles in key aroma-related compound classes, including higher alcohols, esters, and terpenes. Regarding wine type, dry red and dry white wines generally showed higher overall concentrations of aroma compounds compared with other wine styles. Geographical distribution analysis further indicated that samples from China, Australia, Croatia, Spain, and Hungary contained the richest volatile compound profiles. From a methodological perspective, liquid–liquid extraction coupled with gas chromatography–mass spectrometry (LLE-GC–MS), solid-phase extraction combined with gas chromatography–mass spectrometry-olfactometry (SPE-GC–MS-GC-O), and solid-phase microextraction coupled with gas chromatography–mass spectrometry (SPME-GC–MS) captured broader sets of aroma compounds in the assembled records. Taken together, these descriptive patterns may reflect biological variation, sampling imbalance and differences in analytical coverage; the factors were not jointly modelled.

Based on the systematic analysis of 103 studies, the established database contained a total of 792 aroma compounds. In terms of chemical classification, esters (34%), higher alcohols (27%), aldehydes and ketones (17%), organic acids (11%), other compounds (9%), and terpenes (2%) constituted the major classes of wine aroma compounds (Fig. 5B). On this basis, qualitative visualization analysis further revealed the effects of grape variety, wine type, geographical origin, and analytical method on the composition of volatile compounds. The results showed that Muscat, Merlot, Marselan, and Cabernet Sauvignon accounted for the highest proportions of identified compounds, indicating broad compound coverage in the assembled records. In terms of wine type, dry red and dry white wines contained the richest diversity of aroma compounds, with substantially higher chemical diversity than the other five wine styles included in the dataset. Geographical analysis further showed that wines from China, Spain, Brazil, Australia, and Chile exhibited the highest proportions of identified compounds, reflecting the remarkable aroma diversity of these production regions. At the methodological level, solid-phase extraction coupled with gas chromatography–mass spectrometry (SPE-GC–MS), liquid–liquid extraction coupled with gas chromatography–mass spectrometry (LLE-GC–MS), and headspace solid-phase microextraction coupled with gas chromatography–mass spectrometry (HS-SPME-GC–MS) identified the highest proportions of compounds, reflecting their analytical coverage in the assembled records. Overall, these patterns describe factor-associated heterogeneity without ranking the independent contribution of each factor.

*Note:* The legend represents the classification of aroma *compounds. NS* indicates no significant difference (*p* ≥ 0.05), whereas *** indicates a statistically significant difference *at p* < *0.001.*

### 3.5 Screening of key differential compounds based on multi-model analysis

#### 3.5.1 Identification of varietal characteristic markers

Through systematic literature data integration and OAV calculation, a total of 191 aroma-active compounds with available thresholds (OAV > 1) were screened from the 792 identified volatile compounds. To systematically identify varietal aroma markers, a three-step screening strategy was subsequently applied, including PCA, PLS-DA, and RF modelling(Yavas et al., 2025). This integrated analytical framework provided the basis for the subsequent identification of key varietal aroma compounds.

Unsupervised PCA was performed to analyze the volatile profiles of six major grape varieties (Fig. 6A). The results showed that the first two principal components explained 25.5% (PC1) and 16.8% (PC2) of the total variance, respectively, with a cumulative explained variance of 42.3%. The PCA score plot showed that all wine samples were located within the 95% Hotelling's T^2^ confidence ellipse, indicating the absence of significant outliers in the dataset. Clear within-group clustering and between-group separation were observed in the principal component space. PC1 (25.5%), as the major discriminative dimension, effectively separated the grape varieties into two major groups. Samples of M and Me were tightly clustered on the negative side of the axis, whereas S and Cs samples were mainly distributed on the positive side. This separation may reflect differences in their aroma activity profiles, which may be associated with variations in the biosynthetic capacity of fermentation-derived esters and higher alcohols. PC2 (16.8%) played an important role in capturing secondary aroma characteristics and further discriminating varieties located within the same PC1 quadrant. Along the vertical axis, M and Ma samples were distributed on the positive side of PC2, whereas Me and S samples were clustered on the negative side. The discriminative contribution of PC2 may be attributed to subtle variations in varietal-specific precursor compounds, particularly the differences between terpene-rich aromatic Muscat wines and fatty acid-derived compounds in Merlot and Shiraz wines. The synergistic integration of PC1 and PC2 established a comprehensive chemical fingerprinting space(Chen et al., 2026), enabling effective discrimination of the six grape varieties based on their distinct aroma characteristics.

**Fig. 6A-F:**
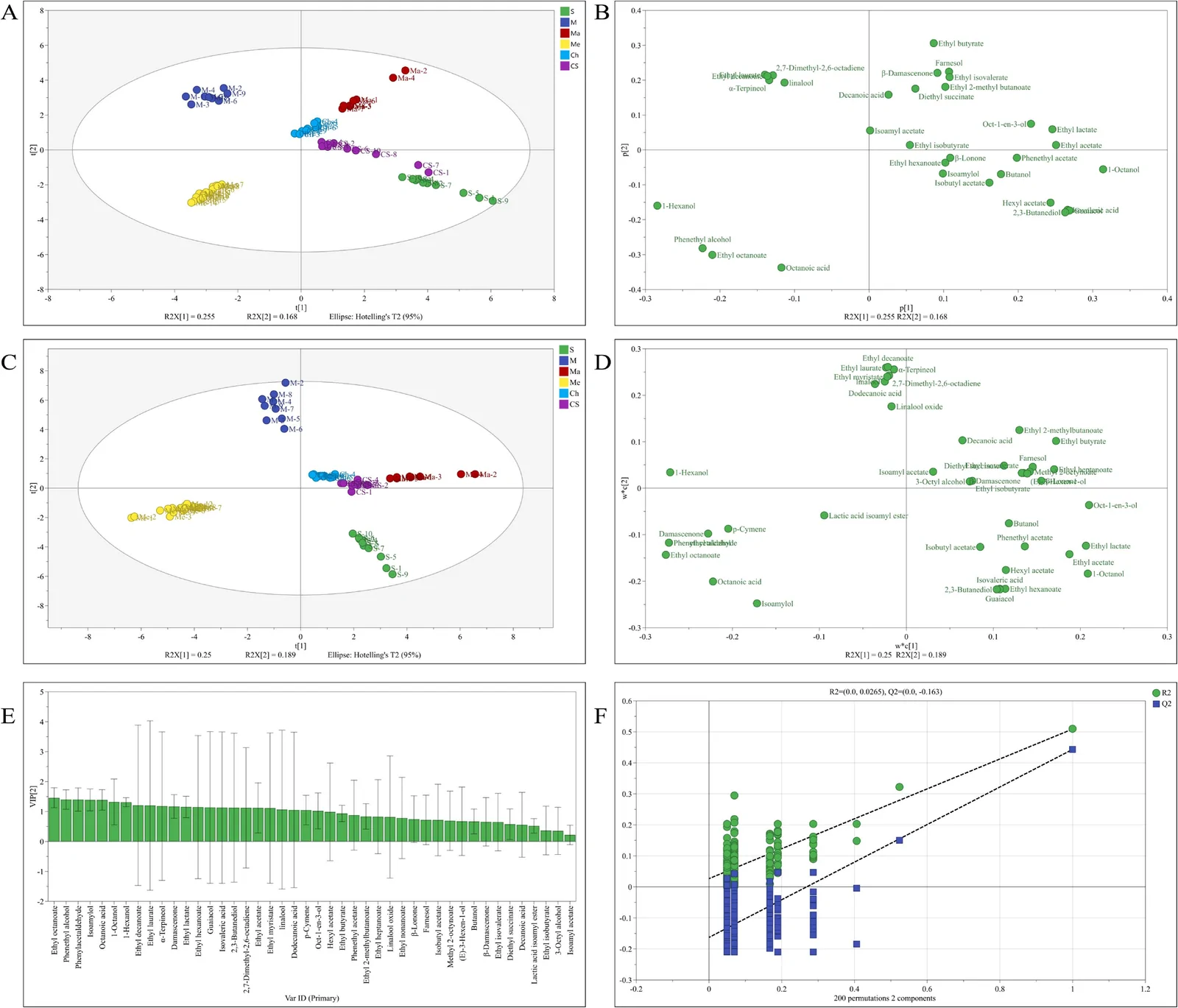
Multivariate analysis of aroma compounds in different wine samples. (A) PCA score plot; (B) PCA loading plot; (C) PLS-DA score plot; (D) PLS-DA loading plot; (E) VIP plot; (F) permutation test plot. (G) Variable importance plot of the random forest model. *Note:* S-Shiraz; M-Muscat; Ma-Marselan; Me-Merlot; Ch-Chardonnay; CS-Cabernet Sauvignon.

The loading plot (Fig. 6B) revealed clear distribution patterns of different volatile compounds in the principal component space. Along the positive direction of PC1, compounds such as ethyl acetate, ethyl lactate, 1-octen-3-ol, and octanol were mainly clustered. These compounds are generally associated with fruity, sweet, and fermentation-derived aroma characteristics. Therefore, the positive axis of PC1 mainly reflects the cumulative contribution of esters and part of the alcohol fraction. Correspondingly, the distribution of S samples in this region suggests that their aroma profiles are predominantly characterized by ester-derived and fermentation-related notes. In contrast, the negative direction of PC1 was mainly associated with compounds such as 2-phenylethanol, octanoic acid, and ethyl octanoate, which are more commonly related to fatty, waxy, or heavier aroma characteristics. This indicates that the negative axis of PC1 represents the contribution of fatty acids and higher alcohol-related compounds, which is consistent with the distribution patterns of M and Me samples Along the positive direction of PC2, key aroma compounds including ethyl butyrate, ethyl hexanoate, ethyl 3-methylbutanoate, β-damascenone, and linalool was highly concentrated. These compounds are characterized by relatively low odor thresholds and are known to contribute significantly to floral and fruity aroma perception(Ferreira, 2010). This suggests that the positive axis of PC2 mainly reflects the contribution of highly aroma-active compounds, which corresponds to the distribution pattern of Ma samples in this region. By contrast, the negative direction of PC2 was mainly associated with compounds such as 1-hexanol, octanoic acid, and 2-phenylethanol, which are often linked to green, grassy, or oily aroma notes and mainly contribute as background or supporting aroma components(Lyu, Ma, Xu, Nie, & Tang, 2019). This distribution pattern was consistent with the positioning of Cs samples in the PCA space.

To further identify varietal aroma markers, a supervised partial least squares discriminant analysis (PLS-DA) model was established. The model extracted two principal components, with cumulative explained variances of R^2^X(cum) = 0.439 and R^2^Y(cum) = 0.603, and a predictive ability of Q^2^(cum) = 0.545, indicating internal model fit and cross-validated predictive performance.

The score plot (Fig. 6C) showed that all samples were located within the 95% Hotelling's T^2^ confidence interval, with no obvious outliers, demonstrating a clear and independent clustering trend among the different grape varieties. The spatial distribution patterns of the varieties were well defined: S and M samples exhibited distinct separation, indicating significant differences in their chemical compositions. Meanwhile, Ma and Me samples were mainly clustered in the upper and lower regions of the score plot, respectively, further confirming the compositional differences among varietal groups.

The loading plot (Fig. 6D) further illustrated the contribution intensity of volatile aroma compounds to the classification performance of the PLS-DA model. Octanol, 1-octen-3-ol, ethyl lactate, ethyl heptanoate, ethyl nonanoate, and ethyl acetate showed the highest contributions to the first principal component. In contrast, isoamyl alcohol, 2,3-butanediol, isovaleric acid, and guaiacol were identified as the key driving factors responsible for the separation along the second principal component.

The PLS-DA model effectively discriminated among the wine samples based on key volatile compounds with VIP values >1.0 (Fig. 6E). These compounds mainly comprised ethyl esters, higher alcohols, and fatty acids, forming the core aroma framework of the investigated wines. Ethyl octanoate (VIP = 1.46) showed the strongest discriminative contribution. As a typical fermentation-derived ester, it is known to impart pleasant fruity, pear-like, and floral aroma characteristics to wine(Ferreira, 2010; Ruiz et al., 2019; Styger, Prior, & Bauer, 2011b). Given its selected odor threshold of 5 μg/L and its high VIP score, the results suggest that variations in lipid metabolism during fermentation may substantially alter the intensity of fruity aroma perception among different groups. Other ester compounds, such as ethyl decanoate and ethyl dodecanoate (VIP > 1.0), further contributed to the complexity of sweet and soapy aroma notes and can serve as important indicators of yeast esterification efficiency. Among higher alcohol- and amino acid-derived compounds, 2-phenylethanol (VIP = 1.40) and 2-phenylacetaldehyde (VIP = 1.40) also showed strong discriminative importance. These compounds are typically produced from phenylalanine through the Ehrlich pathway. 2-Phenylethanol is well recognized for its rose-like floral aroma, whereas 2-phenylacetaldehyde contributes honey-like notes. Their high VIP values indicate that differences in nitrogen utilization and amino acid catabolism by *Saccharomyces cerevisiae* or non-*Saccharomyces cerevisiae* yeasts may vary substantially among different grape varieties(Chatonnet, Dubourdieu, Boidron, & Lavigne, 1993; Legras, Merdinoglu, Cornuet, & Karst, 2007), thereby directly influencing the floral intensity of the final wines.

In addition, C6 alcohols and fatty acids also played important roles in sample discrimination. 1-Hexanol (VIP = 1.31), which is typically associated with herbaceous and green aroma notes, is mainly derived from the lipoxygenase pathway of grape membrane lipids(Pilati et al., 2014). Its identification as a key marker suggests potential differences in grape maturity or pre-fermentation skin maceration treatments among the samples. At the same time, octanoic acid (VIP = 1.39) and isoamyl alcohol (VIP = 1.39), although often associated with fatty or fusel-like notes at high concentrations, act as important precursors for the biosynthesis of corresponding fruity esters. The distribution patterns of these high-VIP markers highlight the complex interactions between varietal precursor composition and fermentation-driven transformation, which together define the distinctive terroir and process-related aroma characteristics of wine. To further evaluate the robustness of the model, a permutation test with 200 iterations was performed (Fig. 6F). The validation results showed R^2^ = 0.0237 and Q^2^ = −0.165. The negative Q^2^ intercept supports the exploratory model relative to permuted labels but does not establish external predictive validity.

In the KNN sensitivity analysis (k = 5), PCA explained 25.57% and 16.72% of variance on PC1 and PC2 (42.29% combined). PLS-DA yielded R^2^X(cum) = 0.436, R^2^Y(cum) = 0.379 and Q^2^(cum) = 0.323. The similar predictor-space variance but lower R^2^Y and Q^2^ indicate that supervised separation was sensitive to missing-data handling; these results do not establish the stability of the 31-marker panel.

To complement the linear discriminant model (PLS-DA) with a nonlinear approach, an RF model was introduced for exploratory screening of the 80-variable RF input matrix. The contribution of each variable to varietal discrimination was evaluated using the percentage increase in mean squared error (%IncMSE) as the variable importance metric(Athanasiadis & Ioannides, 2021).

The mean five-fold cross-validation accuracy was 0.870. The OOB confusion matrix contained 60 correct classifications among 63 samples (accuracy = 0.952); one CS and two S samples were classified as Ch (Supplementary Table S7). These are internal estimates, and independent external test accuracy was not established.

The top 50% of variables ranked by the RF model were retained for comparison with compounds selected by PCA (absolute loading value >0.5) and PLS-DA (VIP > 1.0) (Fig. 6G). The intersection of the three screening sets contained 31 compounds. Highly ranked compounds included ethyl octanoate, ethyl 2-methylbutanoate, isoamyl alcohol, and 2-phenylethanol. Agreement across models fitted to the same dataset provides exploratory support for variable prioritization but does not establish external robustness.

**Fig. 6G:**
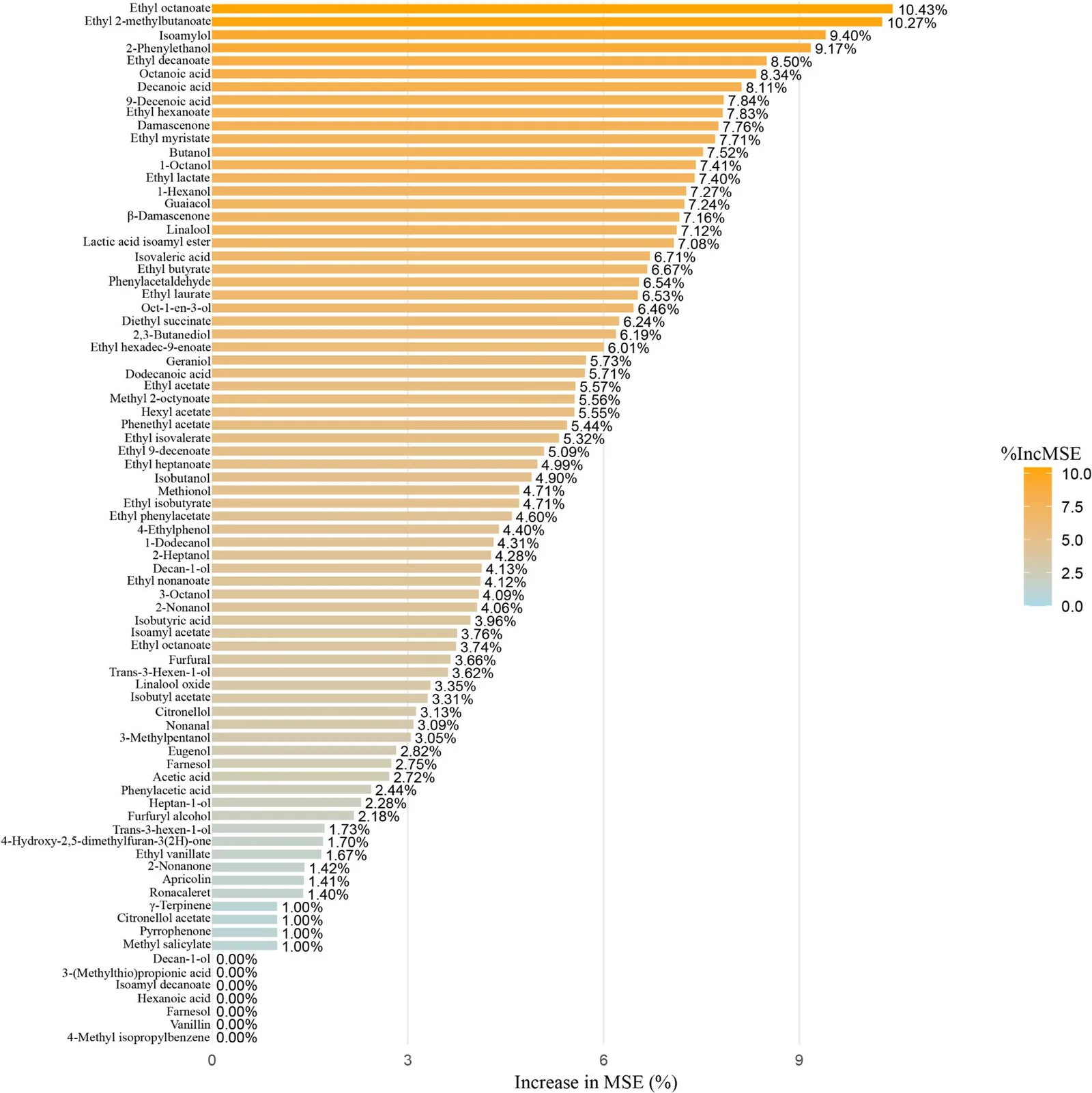
Variable importance ranking of the random forest model based on %IncMSE.

To assemble a candidate set of varietal markers, an intersection-based screening strategy was applied across the three analytical models. A compound was retained only if it simultaneously met all three criteria: a VIP value >1 in PLS-DA, a top-50% ranking in RF permutation importance, and an absolute PCA loading value >0.5. A total of 31 compounds met these criteria and formed the final candidate panel (Table 1). The panel is exploratory and does not establish external predictive validity.

**Table 1:** Varietal aroma compounds retained by the intersection of PCA, PLS-DA and RF screening.

No.	CAS No.	Compound	Selection criteria	Odor description	Concentration (μg/L, mean ± SD)	Quantification method	Threshold (μg/L)	OAV
1	111–87-5	1-Octanol	P, V, R	Fruity	23.04 ± 7.96	Absolute	120 (Ref-087)	1.884
2	111–27-3	1-Hexanol	P, V, R	Herbaceous	107.36 ± 35.54	Absolute	1100 (Ref-096)	51.521
3	71–36-3	Butanol	P, V, R	Pungent	127.89 ± 4.778	Absolute	150,000 (Ref-096)	1.265
4	3391-86-4	Oct-1-en-3-ol	P, V, R	Floral	59.62 ± 21.84	Absolute	20 (Ref-094)	6.395
5	78–70-6	Linalool	P, V, R	Floral	0.45 ± 0.11	Absolute	15 (Ref-092)	147.367
6	7452-79-1	Ethyl 2-methylbutanoate	P, V, R	Fruity	73.64 ± 39.658	Absolute	1 (Ref-095)	737.64
7	123–51-3	Isoamylol	P, V, R	Pungent	2229.10 ± 148.02	Absolute	30 (Ref-061)	74.303
8	60–12-8	Phenethyl alcohol	P, V, R	Floral	2666.89 ± 1220.34	Absolute	1100 (Ref-025)	2.424
9	106–33-2	Ethyl laurate	P, V, R	Fruity	543.031 ± 57.041	Absolute	500 (Ref-005)	1.094
10	110–19-0	Isobutyl acetate	P, V, R	Fruity	1842.75 ± 123.47	Absolute	1600 (Ref-096)	1.152
11	111–11-5	Methyl octanoate	P, V, R	Fruity	7.89 ± 0.65	Absolute	4 (Ref-005)	1.973
12	141–78-6	Ethyl acetate	P, V, R	Fruity	10,812.79 ± 1355.31	Absolute	7500 (Ref-096)	1.442
13	142–92-7	Hexyl acetate	P, V, R	Fruity	2.28 ± 1.99	Absolute	1.5 (Ref-030)	1.52
14	103–45-7	Phenethyl acetate	P, V, R	Fruity	254.28 ± 56.19	Absolute	250 (Ref-095)	1.017
15	106–24-1	geraniol	P, V, R	Floral	595.97 ± 33.386	Semi-quantitative	30 (Ref-092)	19.866
16	97–64-3	Ethyl lactate	P, V, R	Fruity	17,453.80 ± 855.29	Absolute	14,000 (Ref-096)	1.247
17	98–55-5	α-Terpineol	P, V, R	Fruity	307.72 ± 12.23	Absolute	250 (Ref-005)	1.231
18	110–38-3	Ethyl caprate	P, V, R	Caramel	690.31 ± 335.92	Absolute	200 (Ref-096)	3.452
19	105–54-4	Ethyl butyrate	P, V, R	Fruity	65.37 ± 20.99	Absolute	20 (Ref-096)	3.269
20	123–25-1	Diethyl succinate	P, V, R	Fruity	24,787.696 ± 135.28	Absolute	6000 (Ref-073)	4.131
21	108–64-5	Ethyl isovalerate	P, V, R	Fruity	47.12 ± 0.37	Absolute	3 (Ref-095)	15.707
22	513–85-9	2,3-Butanediol	P, V, R	Caramel	849,623.1 ± 9484.406	Semi-quantitative	120,000 (Ref-096)	7.080
23	97–62-1	Ethyl isobutyrate	P, V, R	Fruity	1181.04 ± 54.939	Absolute	15 (Ref-026)	78.736
24	106–32-1	Ethyl caprylate	P, V, R	Fruity	2041.31 ± 712.91	Absolute	5 (Ref-096)	408.262
25	334–48-5	Decanoic acid	P, V, R	Animal	1457.12 ± 36.16	Absolute	1000 (Ref-096)	1.457
26	503–74-2	Isovaleric acid	P, V, R	Animal	237.34 ± 15.31	Absolute	33 (Ref-092)	7.192
27	79–31–2	Isobutyric acid	P, V, R	Caramel	3675.049 ± 260.04	Absolute	2300 (Ref-096)	1.598
28	124–07-2	Octanoic acid	P, V, R	Animal	1532.79 ± 68.72	Absolute	500 (Ref-096)	3.066
29	23,726–93-4	β-Damascenone	P, V, R	Fruity	19.31 ± 5.67	Absolute	0.05 (Ref-092)	386.2
30	90–05-1	Guaiacol	P, V, R	Pungent	28.87 ± 10.242	Absolute	23 (Ref-092)	1.26
31	123–66-0	Ethyl hexanoate	P, V, R	Fruity	442.49 ± 68.13	Absolute	14 (Ref-095)	31.606

Note: Compound-specific threshold sources are shown as Reference IDs in the threshold column; corresponding source records, matrices and protocols are listed in Supplementary Table S2, with complete references in Table S4. OAVs are comparative estimates based on selected literature thresholds, not matrix-independent sensory constants.

V: VIP > 1; P: absolute PCA loading >0.5; R: Random forest importance ranking top 50%. All three criteria were required simultaneously; P, V, R indicates inclusion in their intersection.

### 3.6 Proof-of-concept evaluation of the literature-derived aroma-marker panel in commercial wines

To further evaluate the applicability of the literature-derived aroma-marker panel to commercial wines, 24 Cabernet Sauvignon and Marselan samples (12 per variety) were subjected to a proof-of-concept evaluation. As shown in Fig. 7, the two grape varieties were clearly separated along the first principal component in the PLS-DA score plot, with no overlap observed between the two groups. All samples were located within the corresponding 95% Hotelling's T^2^ confidence ellipses. The PLS-DA model yielded R^2^X(cum) = 0.560, R^2^Y(cum) = 0.971 and Q^2^(cum) = 0.947; ROC analysis yielded an AUC of 0.840. These are internal estimates within the analyzed cohort. No independent external test cohort was available, and independent test-set accuracy was not estimated. The small two-cultivar sample supports an initial comparison, while transferability across other cultivars, regions, vintages and wine styles requires independent validation.

**Fig. 7:**
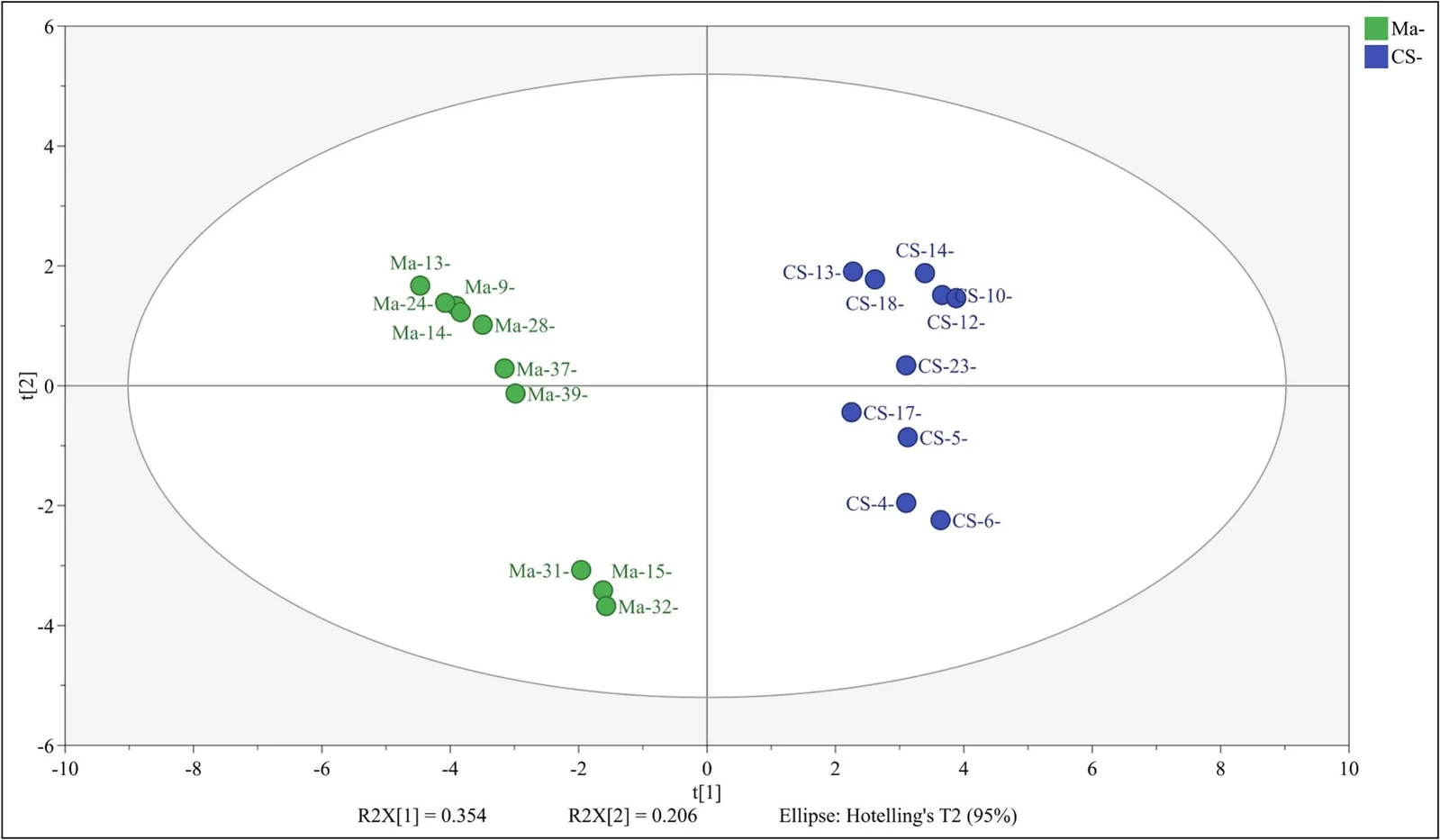
PLS-DA score plot of the proof-of-concept model based on calibrated or internal-standard semi-quantitative measurements (Supplementary Table S6). *Note:* Ma-, experimentally measured Marselan samples; CS-, experimentally measured Cabernet Sauvignon samples.

### 3.7 Sensory characterization of cabernet sauvignon and Marselan wines

#### 3.7.1 Descriptive sensory analysis

The intensity of key aroma attributes was evaluated by a panel of 10 trained assessors using a 0–5 scoring scale. The sensory evaluation results of the experimental wine samples, together with the typical sensory descriptors reported in the literature, are summarized in Table 2. The experimental sensory profiles were compared qualitatively with varietal characteristics described in previous studies. Cabernet Sauvignon wines are characterized by pronounced fruity and green aroma attributes(Zhang et al., 2021), whereas Marselan wines are distinguished by higher intensities of floral and red-fruit aroma notes. Literature-derived numerical scores were removed from Table 2 because the source scales, descriptors and panel designs were not sufficiently comparable; the qualitative descriptors were retained.

**Table 2:** Comparison of sensory characteristics between experimental samples and literature data for Cabernet Sauvignon and Marselan wines.

Variety	Data source	Fruity	Floral	Caramel	Animal	Green	Spicy	Overall typical descriptors
Cabernet Sauvignon	Experimental data	3.48 ± 0.53	3.13 ± 0.38	2.83 ± 0.54	1.47 ± 0.15	3.23 ± 0.47	2.19 ± 0.30	Blackcurrant, black fruit, green pepper, herbaceous notes
	Literature data	–	–	–	–	–	–	Dominated by blackcurrant and berry aromas, often accompanied by vegetal notes
Marselan	Experimental data	3.45 ± 0.43	3.31 ± 0.51	3.19 ± 0.40	1.64 ± 0.39	2.43 ± 0.43	2.21 ± 0.36	Floral, red fruit, dried flower, cherry, violet aromas
	Literature data	–	–	–	–	–	–	Characterized by intense red fruit and distinctive floral notes

Note: — indicates that heterogeneous literature-derived sensory scores were not pooled; literature descriptors are retained for qualitative comparison only.

The sample effect was significant for fruity (F23 = 17.95), floral (11.86), caramel (9.21), animal (7.18), green/herbaceous (14.37) and spicy/pungent (10.72) attributes (all *p* < 0.001; Table S5A). PanelCheck identified sample discrimination for 8–9 of 10 assessors, duplicate-evaluation MSE values of 0.31–0.43, and RV coefficients of 0.69–0.79 (Tables S5B–S5C), indicating residual panelist-related variation despite sample discrimination and repeatability.

## 4 Discussion

### 4.1 Construction of wine aroma database derived from literature

In this study, a literature-derived wine aroma database was established by integrating quantitative volatile compound information from large-scale published studies, providing a systematic framework for reusing previously generated aroma datasets. Unlike conventional aroma studies based on limited experimental samples, this database incorporated aroma profiles reported from 103 studies involving 792 volatile compounds, 38 grape cultivars, and 22 countries, thereby expanding the understanding of global wine aroma diversity. The database covers major volatile compound categories, including esters, higher alcohols, aldehydes, organic acids, ketones, terpenes, and other aroma-related compounds, which represent major chemical contributors to wine sensory characteristics(Cosme, Vilela, Oliveira, Aires, & Pinto, 2025; Zheng, Sun, Zhao, Zheng, & Li, 2016).

The geographical and varietal coverage of the database also reflected the diversity of global wine research. The included studies involved major wine-producing regions, including China, Spain, Australia, Brazil, the United States, and European countries, while representing important cultivars such as Cabernet Sauvignon, Merlot, Chardonnay, Muscat, Marselan, and Shiraz. This large-scale integration provides a broader perspective for understanding how genetic background, cultivation region, winemaking practices, and analytical approaches collectively influence wine aroma composition(Leeuwen et al., 2024; Wei-Peng et al., 2018).

Compared with traditional experimental datasets generated under identical analytical conditions, literature-derived databases have the advantage of capturing global variability and long-term research accumulation. However, the establishment of such databases requires systematic harmonization of compound identities, concentration units, analytical metadata, and odor thresholds(Fryer, Sartori, Thrall, & Tomasino, 2025). In this study, chemical identities were standardized using chemical identifiers, and quantitative information was converted where sufficient information was available, enabling cross-study comparisons and subsequent chemometric analysis. Therefore, this database represents not only a collection of volatile compounds but also a structured resource for future aroma prediction, varietal authentication, and intelligent wine quality evaluation.

### 4.2 Relationship between the 31 candidate marker compounds and grape varietal characteristics

Based on integrated chemometric analysis combining PCA, PLS-DA, and random forest models, 31 volatile compounds were identified as candidate varietal markers(Cosme, Vilela, Oliveira, Aires, & Pinto, 2025). These compounds were retained in the intersection of PCA, PLS-DA and RF screening, with each satisfying all three model-specific criteria; agreement on the same dataset does not establish external validity. The identification of these markers suggests that varietal aroma characteristics are not determined by individual compounds but by coordinated differences among multiple volatile compound networks.

The 31 candidates can be grouped by predominant enological origin, while recognizing overlapping pathways. Grape- or precursor-associated compounds include linalool, geraniol, α-terpineol, 1-hexanol and β-damascenone. Most candidates, including ethyl and acetate esters, higher alcohols, 2,3-butanediol and volatile acids, are associated with fermentation and subsequent transformations. Guaiacol, 1-octen-3-ol and 1-octanol may also reflect processing, microbial activity, oxidation or maturation. Methyl octanoate is a fatty-acid methyl ester, but its specific formation pathway was not established in this study. Shared fermentation pathways, variable precursor availability, frequent analytical detection and available odor thresholds may explain selection of these classes; these are plausible interpretations rather than pathways directly tested here.

The database analysis showed an unadjusted association between grape variety and volatile composition; wine type, geography, analytical methodology and study identity were not jointly controlled. Significant differences were observed among cultivars, particularly in major aroma-related compound classes such as higher alcohols, esters, and terpenes. This finding supports the concept that grape genetic background provides the fundamental basis for varietal aroma expression(Leeuwen et al., 2024).

The potential relevance of these candidate markers was explored using Cabernet Sauvignon and Marselan wines. These two cultivars were selected because they were among the most frequently investigated varieties in the database and showed distinct aroma characteristics. Cabernet Sauvignon is generally associated with characteristic green, herbaceous, and black-fruit sensory attributes, whereas Marselan exhibits stronger floral and red-fruit-related aroma expression. The chemical and sensory profiles provide preliminary descriptive evidence; they do not establish direct compound–sensory relationships or transferable varietal prediction.

Nevertheless, these compounds should not be considered as absolute “fingerprints” of specific cultivars. Wine aroma formation is influenced by multiple interacting factors, including climate, vineyard management, fermentation microorganisms, maturation conditions, and analytical methodology(Ciupeanucalugaru, Dodocioiu, Buzatu, & Fang, 2026; Leeuwen et al., 2024). Therefore, the 31 compounds should be regarded as candidate indicators reflecting varietal tendencies rather than independent determinants of aroma identity.

The commercial-wine experiment included only 12 Cabernet Sauvignon and 12 Marselan wines and should be interpreted as proof of concept. Separation within this fitted model does not establish universal discrimination across other cultivars, regions, vintages or styles. Larger independent cohorts measured under harmonized analytical conditions are needed.

### 4.3 Fragmentation of wine aroma databases and the necessity of standardized data output

Although considerable progress has been achieved in wine aroma research, existing aroma datasets remain highly fragmented. Independent studies differ substantially in sample preparation, extraction procedures, instrumental platforms, compound identification criteria, quantification strategies, and reporting formats, which limits direct comparison and secondary utilization of published data(Pinu et al., 2019). For example, compound concentrations may be reported as absolute concentrations, relative abundances, or semi-quantitative values, while non-detected and unreported compounds are often inconsistently defined.

Min–Max normalization and *Z*-score standardization have similar roles in rescaling variables, but neither, when applied to pooled data, explicitly estimates or removes study-specific technical effects. No study-level batch correction was applied here. Residual extraction and instrumental differences may affect PCA loadings, PLS-DA VIP scores and RF rankings, particularly where cultivar and analytical method are confounded, and may change which compounds enter their intersection. Agreement among the three models fitted to the same dataset does not remove this shared source of bias. Its magnitude and the stability of the 31-marker panel under alternative scaling were not quantified. Further assessment should use alternative scaling, method-stratified analyses, leave-one-study-out validation and independent measurements.

Odor thresholds depend on matrix composition, ethanol, temperature and sensory protocol, so heterogeneous literature thresholds introduce uncertainty, especially near OAV = 1. Only 191 of 792 compounds had usable thresholds; exclusion of the other 601 may favour historically well-studied volatiles. Threshold-missing compounds may still have sensory or varietal relevance. The marker panel is conditional on threshold availability and requires matrix-matched sensory confirmation.

Unequal coverage of cultivars and regions may weight marker selection toward mainstream commercial varieties and heavily studied regions. Publication frequency and analytical attention may influence the panel, whose applicability to niche cultivars remains untested. The KNN sensitivity results also show that supervised separation depends on missing-data handling; zero coding can conflate analytical non-coverage with true absence.

Sample-level paired chemical and sensory records were insufficient to reconstruct and verify all compound–attribute correlations. The Pearson network and its associated interpretations were therefore removed. Individual compounds are not assigned direct sensory contributions from these data. Future work should retain complete paired measurements, report coefficients and exact *p*-values, and correct for multiple testing.

Compound-specific calibration was available for some commercial-wine measurements, whereas others were semi-quantitative estimates relative to the internal standard. Concentrations within calibration ranges do not establish analytical sensitivity, matrix recovery or overall accuracy. LOD/LOQ and spike recovery were not determined, which limits quantitative interpretation.

The fragmentation problem represents one of the major barriers preventing the transformation of accumulated scientific data into reusable digital resources. Statistical normalization reduces numerical scale differences but does not itself correct systematic differences caused by analytical platforms and experimental designs. Therefore, future wine aroma research should move beyond isolated experimental datasets and establish globally compatible standards for data generation and reporting.

From a technical perspective, standardized output formats should include unified compound identification systems, complete sample provenance information, standardized analytical metadata, clear definitions of missing values, and consistent quantitative reporting methods. Such standards would improve data interoperability and enable more reliable applications of machine learning and artificial intelligence in aroma prediction and authentication(Jimenez-Carvelo, Gonzalez-Casado, Gracia Bagur-Gonzalez, & Cuadros-Rodriguez, 2019).

From a policy perspective, international organizations and wine research institutions should promote standardized guidelines for aroma analysis and data sharing. Establishing open-access databases with quality control mechanisms would reduce redundant data generation, improve research efficiency, and facilitate global collaboration Wilkinson, Dumontier, Aalbersberg, Appleton, and Mons (2016).

The complete database has not been deposited in a public repository. Confidential availability to the editor and reviewers limits independent reuse and does not constitute full repository-based FAIR availability or an openly accessible international data standard.

### 4.4 Future perspectives: integration of standards, policies, and intelligent technologies

The development of standardized wine aroma databases provided an important foundation for the intelligent transformation of the wine industry. In the future, standardized chemical datasets combined with machine learning algorithms may enable rapid prediction of wine sensory characteristics, cultivar authentication, and quality evaluation (Fuentes et al., 2020)

However, achieving this goal requires coordinated development in three aspects. First, standardized data generation should be strengthened through unified analytical protocols and reporting frameworks, ensuring that newly generated datasets can be integrated into existing databases. Second, policy support and international cooperation are needed to encourage data sharing, establish reference databases, and develop industry-wide standards(Sidorkiewicz, Krlikowska, Dyczek, Pijet-Migon, & Dubel, 2025). Third, advanced computational technologies, including machine learning, artificial intelligence, and multi-omics integration, should be further applied to uncover complex relationships between chemical composition and sensory perception(Perrot, Roche, Tonda, Lutton, & Thomas-Danguin, 2023).

Overall, this study provides a preliminary framework demonstrating the potential of literature-derived databases for identifying varietal aroma markers. Future efforts should focus on transforming fragmented aroma information into standardized, expandable, and intelligent data resources, thereby supporting evidence-based decision-making and technological innovation in the global wine industry.

## 5 Conclusions

This study established a literature-derived wine aroma database by harmonising published volatile data from 103 studies across 792 compounds, 38 grape cultivars, and 22 countries. By integrating compound identities, quantitative information, analytical metadata, sample provenance, and odor thresholds, the resulting dataset provides a structured basis for the secondary use and cross-study comparison of previously fragmented wine aroma data.

The intersection of PCA, PLS-DA and random forest screening identified a panel of 31 candidate compounds associated with varietal differentiation. Their potential relevance was explored through chemical and sensory evaluation of 24 commercial Cabernet Sauvignon and Marselan wines. Internal discrimination (R^2^Y = 0.971, Q^2^ = 0.947; AUC = 0.840) requires confirmation in an independent cohort. Nevertheless, these compounds should be regarded as candidate indicators of varietal tendencies rather than universal chemical fingerprints, because their abundance may also be affected by vintage, region, winemaking practice, and analytical platform. Cross-study heterogeneity, missing-value handling and threshold coverage remain limitations. Future work should prioritize harmonized measurements, matrix-matched thresholds and independent validation.

## CRediT authorship contribution statement

**Mei-Ling Yang:** Writing – original draft, Investigation, Formal analysis. **Hui Wang:** Writing – original draft, Investigation, Formal analysis. **Li-Xia Zhu:** Writing – review & editing, Project administration, Funding acquisition.

## Ethics statement

This study received ethical approval from the scientific and technological development of Tarim University (9B20250415001). All participants provided written informed consent prior to the study, confirming their voluntary participation and understanding of data usage. All personal data were anonymized, and the study protocols were conducted in strict accordance with the ethical standards for participant rights and privacy protection outlined in the Declaration of Helsinki.

## Declaration of generative AI and AI-assisted technologies in the writing process

During the preparation of this work, the authors used Deepseek to check grammar and spelling. After using this tool, the authors reviewed and edited the content as needed and took full responsibility for the content of the published article.

## Declaration of competing interest

The authors declare that they have no known competing financial interests or personal relationships that could have appeared to influence the work reported in this paper.

## Data Availability

The data that has been used is confidential.
