# Supplementary material for: Quantitative mining of wine aroma literature data for specification reuse
Source: Food Chem X. 2026 Oct 15;40:104611. doi: 10.1016/j.fochx.2026.104611 (PMC13742478; doi:10.1016/j.fochx.2026.104611)
Supplement: Supplementary file 1 [file mmc3.docx]

**Table S7. Out-of-bag confusion matrix for the final random forest model.**

| **Actual / predicted** | **CS** | **Ch** | **M** | **Ma** | **Me** | **S** | **Class total** | **Class recall** |
| --- | --- | --- | --- | --- | --- | --- | --- | --- |
| CS | 9 | 1 | 0 | 0 | 0 | 0 | 10 | 0.900 |
| Ch | 0 | 9 | 0 | 0 | 0 | 0 | 9 | 1.000 |
| M | 0 | 0 | 9 | 0 | 0 | 0 | 9 | 1.000 |
| Ma | 0 | 0 | 0 | 8 | 0 | 0 | 8 | 1.000 |
| Me | 0 | 0 | 0 | 0 | 17 | 0 | 17 | 1.000 |
| S | 0 | 2 | 0 | 0 | 0 | 8 | 10 | 0.800 |

Note: Rows indicate actual classes and columns indicate OOB-predicted classes. Overall OOB accuracy = 60/63 = 0.952. The mean five-fold stratified cross-validation accuracy was 0.870. Balanced bootstrap sampling was applied within each tree to address class imbalance.
